# Marital status and survival of patients with colorectal signet ring cell carcinoma: a population-based study

**DOI:** 10.1038/s41598-020-74720-7

**Published:** 2020-10-21

**Authors:** Li Feng, Yong-jing Yang, Juan Du, Yong-jiang Yu, Jian-dong Diao

**Affiliations:** 1 Department of Radiation Oncology, China–Japan Union Hospital of Jilin University, Changchun, Jilin China; 2 Department of Radiation Oncology, Jilin Cancer Hospital, Changchun, Jilin China; 3 Department of Medical Oncology, Jilin Cancer Hospital, Changchun, Jilin China; 4 Department of Endocrinology, Affiliated Hospital of Changchun University of Traditional Chinese Medicine, Changchun, Jilin China; 5 Department of Oncology and Hematology, China–Japan Union Hospital of Jilin University, Changchun, Jilin China

**Keywords:** Cancer, Oncology, Risk factors

## Abstract

The prognostic role of marital status on colorectal signet ring cell carcinoma (SRCC) has not been studied. In this study, the correlation of marital status with prognosis of colorectal SRCC was analyzed. Eligible subjects were extracted from the Surveillance, Epidemiology, and End Results (SEER) dataset from 2004 to 2015, followed by comparison of cancer-specific survival (CSS) and overall survival (OS) between married and unmarried group. 3152 patients were identified including 1777 married patients (56.38%). Married populations tended to be more patients aged < 65, male, receiving chemotherapy, and less black race and large tumor size compared to unmarried group (all *P* < 0.05).Moreover, 5-year CSS (30.04% vs. 28.19%, *P* = 0.0013) and OS rates (26.68% vs. 22.94%, *P* < 0.0001) were superior in married population. Multivariate analysis revealed that marital status was an independent favorable prognostic indicator, and married population had better CSS (HR: 0.898; 95% CI: 0.822–0.980; *P* = 0.016) and OS (HR: 0.898; 95%CI: 0.827–0.975; *P* = 0.011).In addition, CSS as well as OS were superior in married populations than unmarried ones in most subgroups. Marital status was an independent prognostic factor for survival in patients with colorectal SRCC. Additionally, married patients obtained better survival advantages.

## Introduction

Colorectal cancer (CRC) is the second leading cause of cancer-associated mortality in the USA, also greatly threatening the global health^1^. Despite the diverse subtypes of CRCs, accumulative attention has been recently paid to colorectal signet ring cell carcinoma (SRCC), which was initially proposed by Saphir as well as Laufman in 1951^2^. Stomach is considered to be the most common organ for primary SRCC, while colorectal SRCC is less frequent^3^. Colorectal SRCC is a very rare and special type of CRCs, accounting for only 0.3 to 4.6% of all types of CRCs^4–8^.

The prognostic factors of colorectal SRCC have intensively explored, mostly including clinicopathological features as well as therapeutic strategies^9–12^. However, the present attention has also been paid to social factors, which might be involved in disease progression^13,14^. Among them, marital status as an important social factor has attracted more and more attention. To be specific, marital status has been identified as an independent prognostic indicator in several types of malignancies, including colorectal cancer, pancreatic, breast, lung and prostate cancer, with superior survival in married population^15–19^. However, there is no study concerning the role of marital status on colorectal SRCC survival specially.

The National Cancer Institute (NCI)’s Surveillance, Epidemiology and End Results (SEER) database reports data from 18 population-based cancer registries by covering nearly 30% of the US population^20^. Therefore, we could investigate the correlation of marital status with survival in rare tumors by extracting data from SEER^14,18,21^. This research was designed to examine the association of marital status with survival in colorectal SRCC patients by utilizing SEER database.

## Materials and methods

### Ethics statement

For acquisition of relevant data from the database, we signed the SEER Research Data Agreement (No. 19817-Nov2018) and further searched for data according to the approved guidelines. All extracted data were publicly accessible and de-identified, and data analysis was considered to be non-human subjects by Office for Human Research Protection, therefore, no approval was requested by institutional review board.

### Study population

SEER*State v8.3.6 (released on August 8, 2019) was employed to select and identify qualified subjects, which includes 18 SEER regions during the period of 1998–2015 (2018 submission). The inclusion criteria were as follows: (1) it should be primary colorectal SRCC patients; (2) the diagnosis of SRCC was based on ICD-O-3; coded as 8490/3. Patients were excluded if they had: (1) more than one primary malignancies; (2) reported diagnosis source from autopsy or death certificate or without pathological diagnosis; (3) without certain necessary clinicopathological data, including: AJCC stage, surgical style and marital status; (4) without prognostic information. The remaining qualified populations were included.

### Covariates and endpoint

We analyzed the patients’ characteristics according to the following factors: year of diagnosis (2004–2007, 2008–2011, 2012–2015); insured status (uninsured/unknown, any medicaid/insured); age (< 65, ≥ 65); marital status (unmarried, married); gender (female, male); race (black, white or others); primary site (cecum–transverse colon, descending colon–sigmoid, multiple, rectum and unknown); grade (grade I/II, grade III/IV, unknown); tumor size (≤ 5 cm, ˃5 cm, unknown); AJCC stage (stage I, II, III, IV); surgery (no surgery, local tumor excision/partial colectomy, total colectomy), lymph node dissections (none or biopsy, 1–3 regional lymph nodes removed, ≥ 4 regional lymph nodes removed, unknown), chemotherapy (no/unknown, yes), radiotherapy (no/unknown, yes).The widowed or single (never married or having a domestic partner) or divorced or separated patients were classified as unmarried. The primary tumor site was classified as cecum–transverse colon (including the cecum, appendix, ascending colon, hepatic flexure and the transverse colon), descending colon–sigmoid (including the splenic flexure and descending and sigmoid colons), multiple, rectum and unknown. Year of diagnosis was equally divided into 2004–2007, 2008–2011, 2012–2015, which was referred to the previous papers^22,23^. The grouping of the age and tumor size also refers to the published studies^24,25^. In addition, the staging of cancer is based on the 6th edition of AJCC stage system, which adapted to patients in the SEER database with a diagnosis time of 2004–2015.

The endpoint included cancer-specific survival (CSS) and overall survival (OS). The former was defined as the duration from diagnosis to colorectal SRCC-caused death, and the latter was referred to the duration from diagnosis to all-cause death.

### Statistical analyses

Kaplan–Meier (K–M) method was employed to estimate the univariate analysis, followed by log-rank test for assessing the differences of CSS and OS. Notably, if variables had *P* values ≤ 0.1 in univariate analysis, they were incorporated into multivariate Cox proportional hazard analysis. Similarly, Cox regression analysis was also used for stratified analysis. SPSS software (SPSS Inc., Chicago, USA, version 19.0) was used for statistical analysis, and GraphPad Prism 5 was utilized for plotting survival curves as well as generating forest plots. A two-sided *P* < 0.05 indicated statistical significance.

## Results

### Patient features

**Figure1:**
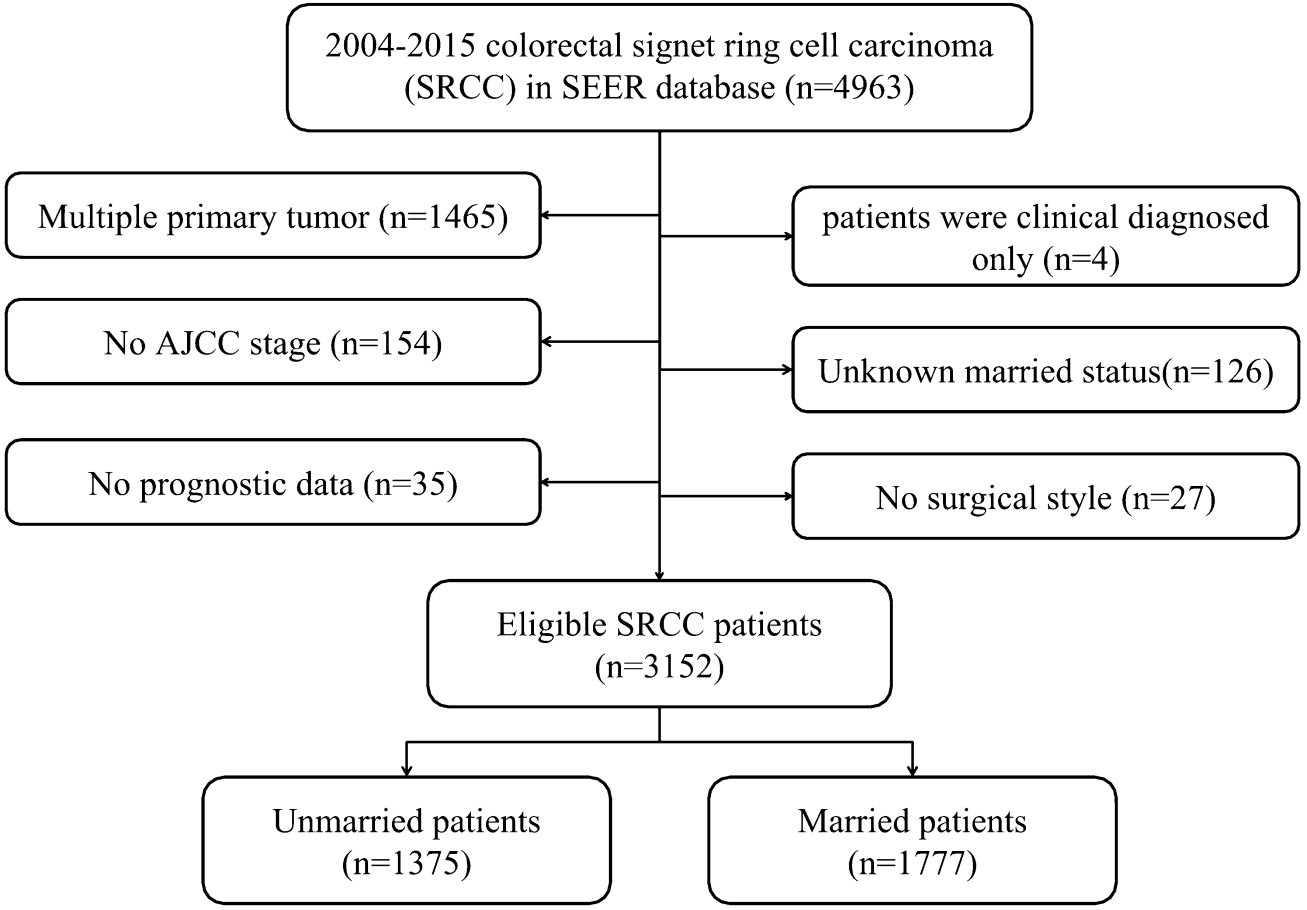
Flow chart of patient selection.

In total, 3152 eligible patients were identified from the SEER database between 2004 and 2015, with a median follow-up duration of 16 months (range: 0–155 months). Afterwards, subjected were categorized into unmarried group (n = 1375, 43.62%) and married group (n = 1777, 56.38%), with specific screening process shown in Fig. 1.Moreover, the baseline characteristics of patients stratified by marital status were summarized in Table 1. To be specific, age (*P* = 0.002), gender (*P* < 0.001), race (*P* < 0.001), tumor size (*P* = 0.002) and chemotherapy (*P* < 0.001) were significantly different between the two groups. Additionally, married populations tended to be more patients aged < 65 (56.95% vs. 48.36%), male (58.98% vs. 41.45%), receiving chemotherapy (63.53% vs. 51.71%), and were less to be black race (7.09% vs. 12.58%) and tumor size ˃5 cm (36.18% vs. 42.04%) in comparison with unmarried ones.

**Table 1:** The clinicopathological characteristics and treatment of the included 3152 colorectal signet ring cell carcinoma patients.

Characteristic	Total	Unmarried	Married	*P*-value
Year at diagnosis				0.068
2004–2007	1066 (33.82%)	440 (32.00%)	626 (35.23%)	
2008–2011	1022 (32.42%)	443 (32.22%)	579 (32.58%)	
2012–2015	1064 (33.76%)	492 (35.78%)	572 (32.19%)	
Insured status				0.405
Uninsured/unknown	969 (30.74%)	412 (29.96%)	557 (31.34%)	
Any medicaid/insured	2183 (69.26%)	963 (70.04%)	1220 (68.66%)	
Age				< 0.001
< 65	1677 (53.20%)	665 (48.36%)	1012 (56.95%)	
≥ 65	1475 (46.80%)	710 (51.64%)	765 (43.05%)	
Gender				< 0.001
Female	1534 (48.67%)	805 (58.55%)	729 (41.02%)	
Male	1618 (51.33%)	570 (41.45%)	1048 (58.98%)	
Race				< 0.001
Black	299 (9.49%)	173 (12.58%)	126 (7.09%)	
White	2584 (81.98%)	1115 (81.09%)	1469 (82.67%)	
Other	269 (8.53%)	87 (6.33%)	182 (10.24%)	
Primary site				0.213
Cecum–transverse colon	1901 (60.31%)	841 (61.16%)	1060 (59.65%)	
Descending colon–sigmoid	493 (15.64%)	206 (14.98%)	287 (16.15%)	
Multiple	48 (1.52%)	28 (2.04%)	20 (1.13%)	
Rectum	623 (19.77%)	262 (19.05%)	361 (20.32%)	
Unknown	87 (2.76%)	38 (2.76%)	49 (2.76%)	
Grade				0.233
Grade I/II	171 (5.43%)	64 (4.65%)	107 (6.02%)	
Grade III/IV	2419 (76.74%)	1067 (77.60%)	1352 (76.08%)	
Unknown	562 (17.83%)	244 (17.75%)	318 (17.90%)	
Tumor size				0.002
≤ 5 cm	1173 (37.21%)	494 (35.93%)	679 (38.21%)	
> 5 cm	1221 (38.74%)	578 (42.04%)	643 (36.18%)	
Unknown	758 (24.05%)	303 (22.04%)	455 (25.60%)	
AJCC stage				0.968
I	147 (4.66%)	64 (4.65%)	83 (4.67%)	
II	444 (14.09%)	192 (13.96%)	252 (14.18%)	
III	1156 (36.68%)	511 (37.16%)	645 (36.30%)	
IV	1405 (44.57%)	608 (44.22%)	797 (44.85%)	
Surgery				0.053
No surgery	650 (20.62%)	287 (20.87%)	363 (20.43%)	
Local tumor excision /Partial colectomy	828 (26.27%)	332 (24.15%)	496 (27.91%)	
Total colectomy	1674 (53.11%)	756 (54.98%)	918 (51.66%)	
Lymph node dissection				0.991
None or biopsy	847 (26.87%)	368 (26.76%)	479 (26.96%)	
1–3	83 (2.63%)	36 (2.62%)	47 (2.64%)	
≥ 4	2222 (70.49%)	971 (70.62%)	1251 (70.40%)	
Chemotherapy				< 0.001
No/unknown	1312 (41.62%)	664 (48.29%)	648 (36.47%)	
Yes	1840 (58.38%)	711 (51.71%)	1129 (63.53%)	
Radiotherapy				0.077
No/unknown	2726 (86.48%)	1206 (87.71%)	1520 (85.54%)	
Yes	426 (13.52%)	169 (12.29%)	257 (14.46%)	

### Marital status and survival

**Figure2:**
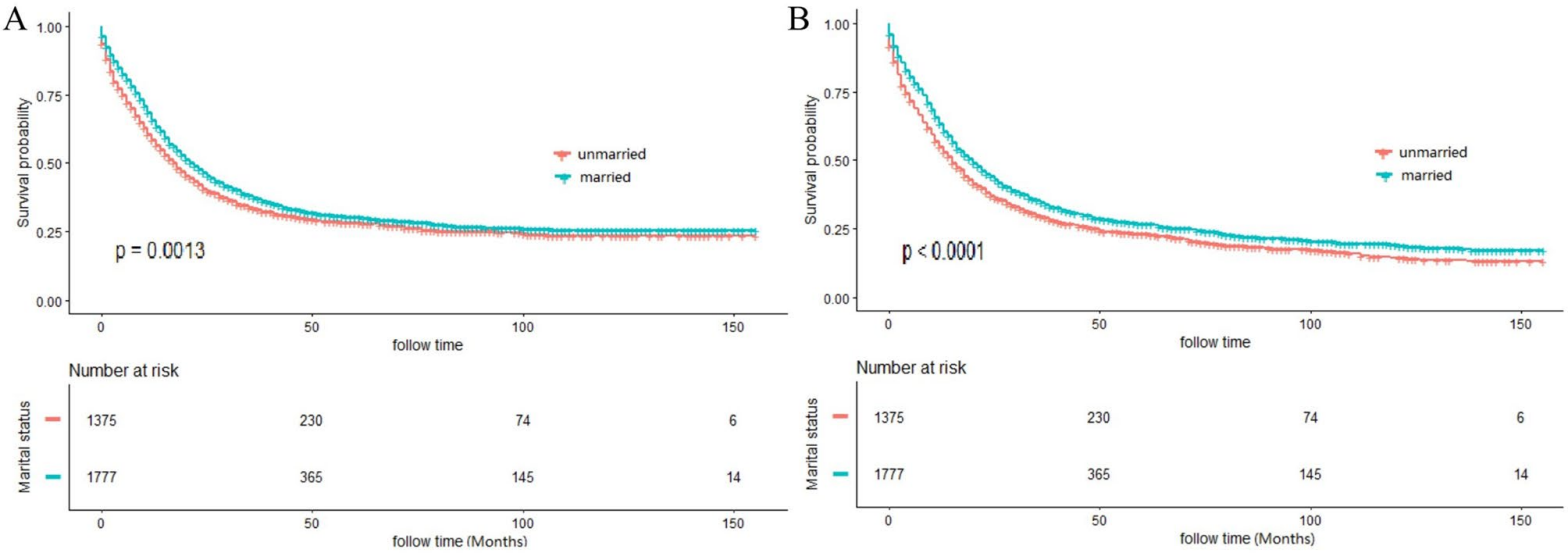
Kaplan–Meier (K–M) curves for cancer-specific survival (CSS) (**A**) and overall survival (OS) (**B**) between unmarried and married patients.

K–M curves revealed significant difference of CSS and OS stratified by marital status (Fig. 2), with superior OS and CSS in married populations than unmarried ones. The 3, 5, 10-year CSS rate was 37.54%, 30.04% and 25.49% in married patients, which was 33.20% ,28.19% and 23.47% in unmarried group (*P* = 0.0013). Meanwhile, the 3, 5, 10-year OS rate was 34.54%, 26.68% and 18.92% in married patients, which was 29.28% ,22.94% and 14.40% in unmarried group (*P* < 0.0001). Univariate log-rank test identified that marital status, primary site, grade, tumor size, AJCC stage, surgery, lymph node dissections and chemotherapy were significantly relate to CSS (*P* < 0.05). After the adjustment of the above variables in the Cox proportional hazards regression model, marital status remained as an independent prognostic indicator, with superior CSS in married populations than unmarried ones (HR: 0.898; 95% CI: 0.822–0.980; *P* = 0.016]. Meanwhile, all aforementioned variables including age and radiotherapy also had significant relationship with OS, and multivariate analysis also found that marital status was a favorable independent prognostic indicator of OS (HR: 0.898; 95%CI: 0.827–0.975; *P* = 0.011). Table 2 showed the detailed results of univariate and multivariate analysis.

**Table 2:** Univariate and multivariate analyses of cancer special survival (CSS) and overall survival (OS) for patients with colorectal SRCC.

Variables	CSS			OS		
	Univariate analysis	Multivariate analysis		Univariate analysis	Multivariate analysis	
	*P* value	HR (95%CI)	*P* value	*P* value	HR (95%CI)	*P* value
Year at diagnosis	0.826		NI	0.962		NI
2004–2007						
2008–2011						
2012–2015						
Insured status	0.443		NI	0.620		NI
Uninsured/unknown						
Any medicaid/insured						
Age	0.731		NI	< 0.001		< 0.001
< 65					Reference	
≥ 65					1.486 (1.359, 1.624)	
Gender	0.176		NI	0.506		NI
Female						
Male						
Race	0.149		NI	0.183		NI
Black						
White						
Other						
Primary site	< 0.001		< 0.001	< 0.001		< 0.001
Cecum–transverse colon		Reference			Reference	
Descending colon–sigmoid		1.196 (1.054, 1.358)	0.005		1.211 (1.074, 1.365)	0.002
Multiple		1.466 (1.050, 2.048)	0.025		1.395 (1.008, 1.930)	0.044
Rectum		1.334 (1.184, 1.503)	< 0.001		1.286 (1.119, 1.477)	< 0.001
Unknown		1.368 (1.075, 1.740)	0.011		1.326 (1.049, 1.675)	0.018
Grade	< 0.001		< 0.001	< 0.001		< 0.001
Grade I/II		Reference			Reference	
Grade III/IV		1.768 (1.417, 2.205)	< 0.001		1.742 (1.426, 2.128)	< 0.001
Unknown		1.763 (1.386, 2.242)	< 0.001		1.739 (1.397, 2.165)	< 0.001
Tumor size	< 0.001		< 0.001	< 0.001		< 0.001
≤ 5 cm		Reference			Reference	
> 5 cm		1.253 (1.128, 1.392)	< 0.001		1.202 (1.091, 1.325)	< 0.001
Unknown		1.301 (1.143, 1.482)	< 0.001		1.294 (1.143, 1.465)	< 0.001
AJCC stage	< 0.001		< 0.001	< 0.001		< 0.001
I		Reference			Reference	
II		1.426 (0.976, 2.083)	0.067		1.552 (1.149, 2.097)	0.004
III		4.794 (3.390, 6.780)	< 0.001		3.921 (2.960, 5.193)	< 0.001
IV		12.086 (8.642, 17.099)	< 0.001		9.649 (7.271, 12.806)	< 0.001
Surgery	< 0.001		< 0.001	< 0.001		< 0.001
No surgery		Reference			Reference	
Local tumor excision/partial colectomy		0.595 (0.491, 0.722)	< 0.001		0.613 (0.510, 0.737)	< 0.001
Total colectomy		0.710 (0.579, 0.871)	0.001		0.745 (0.614, 0.940)	0.003
Dissected lymph node	< 0.001		0.135	< 0.001		0.042
None or biopsy		Reference			Reference	
1–3		0.914 (0.672, 1.244)	0.568		0.913 (0.685, 1.216)	0.325
≥ 4		0.832 (0.693, 1.000)	0.050		0.807 (0.679, 0.959)	0.015
Chemotherapy	0.084		< 0.001	< 0.001		< 0.001
No/unknown		Reference			Reference	
Yes		0.514 (0.467, 0,565)			0.513 (0.467, 0.563)	
Radiotherapy	0.552		NI	0.096		0.132
No/unknown					Reference	
Yes					1.125 (0.965, 1.312)	
Marital status	0.001		0.016	< 0.001		0.011
Unmarried		Reference			Reference	
Married		0.898 (0.822, 0.980)			0.898 (0.827, 0.975)	

*CSS* cancer-specific survival, *OS* overall survival, *NI* not included in the multivariate survival analysis.

### Subgroup analysis on marital status

**Figure3:**
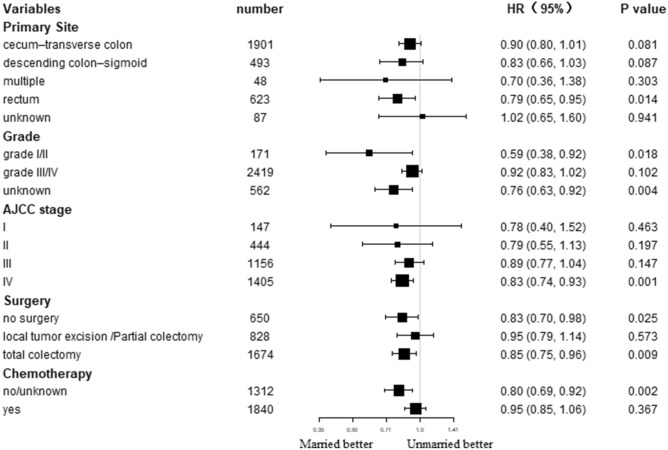
Forest plot of subgroup analysis for cancer-specific survival (CSS).

**Figure4:**
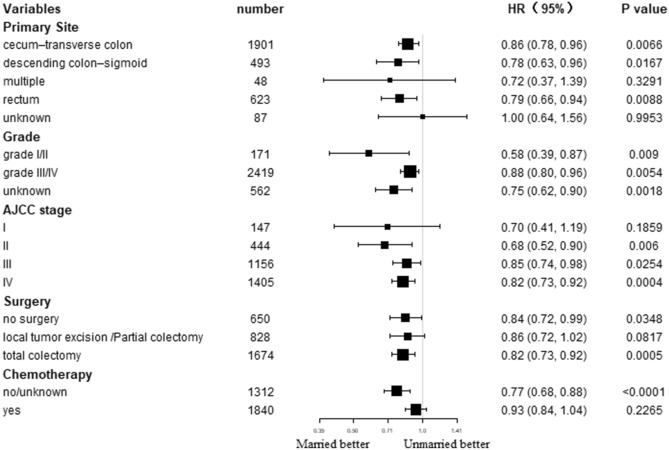
Forest plot of subgroup analysis for overall survival (OS).

The effects of marital status on survival were further determined in different subgroups. Subgroup analysis demonstrated superior OS as well CSS in married populations than unmarried ones in nearly all subgroups (Figs. 3 and 4). Specifically, primary site in rectum, grade I/II, AJCC stage IV, total colectomy, and no/unknown chemotherapy subgroups patients could significantly benefit from married status in terms of CSS (all *P* < 0.05). In addition, most subgroups could significantly benefit from married status in terms of OS (all *P* < 0.05).

## Discussion

To the best of our knowledge, this study is the first to specifically examine whether marital status has a significant impact on the survival of colorectal SRCC patients. By enrolling 3152 colorectal SRCC patients, we observed significantly lower risk of mortality in married populations compared to unmarried ones. After controlling for demographic and tumor characteristics, married populations had a 10.2% decreased death risk compared to unmarried patients with colorectal SRCC. In general, marital status was an independent favorable prognostic factor in colorectal SRCC populations.

Several studies have previously reported the correlation of marital status with prognosis in CRC^26–28^. Xiao et al. found marital status as an independent prognostic indicator in colorectal neuroendocrine neoplasm, with superior OS and CSS in married populations^26^. Furthermore, Ge et al. found worse OS in unmarried populations than married ones in CRC patients with metastasis^27^. By summarizing and analyzing 53 articles concerning the prognosis of CRC, Mozafar SH also confirmed marital status as a prognostic factor for CRC^28^. Taken together, these studies are basically consistent with the results of our study.

In addition to colorectal cancer, marital status has also been found to be significantly associated with prognosis in many other malignancies. For example, Zhou et al. proved that marital status was an independent prognostic risk factor for patients with pancreatic endocrine cancer^18^. Similar findings have also been discovered in breast cancer and nasopharyngeal carcinoma^15,29^. The above researches all exhibited that the prognosis of married patients is remarkably better than that of unmarried ones.

Two potential mechanisms may be used for explanation of the association of marital status with survival. For one thing, married populations have less distress and depression than unmarried patients following tumor diagnosis, because the emotional burden could be shared with their partners, who could also offer proper social support^30,31^. Loneliness and depression can down regulate the cellular immune response^32^, stimulate tumor angiogenesis and increase tumor burden and invasiveness^33–35^. For another thing, married patients with emotional and financial support from their spouses or children could have a better compliance from doctors^36,37^. Thus, they may be more likely to receive active treatments. Similarly, our study found that married patients had a higher rate of receiving chemotherapy. Therefore, social support as well as psychological interventions should be taken into consideration to attenuate the significant survival differences between married and unmarried tumor populations.

However, there are some limitations in this study, which mainly result from the restricted nature of SEER dataset. To begin with, the marital status extracted was recorded at diagnosis. Therefore, it remains unknown whether marital status changed throughout the follow-up, which might influence the outcomes as well. Secondly, the detailed quality of marriage was not available from the database, thereby affecting survival outcomes^38^. Thirdly, detailed therapeutic information is lacking, especially radiotherapy and chemotherapy. Finally, a causal correlation of marital status with survival cannot be proposed due to the research design, which requires further prospective cohort researches for validation. Nevertheless, our findings suggest that marital status has significantly impact on the survival of colorectal SRCC, highlighting the substantial as well as consistent effect of marriage, especially social support, on the detection, therapy and survival of cancer. Moreover, our outcomes also implicate that social support interventions targeting vulnerable populations, including unmarried populations, are likely to greatly enhance the cure probability. These types of interventions might be cost-effective to enhance clinical outcomes among unmarried tumor populations.

## Conclusion

In conclusion, our study found that marital status was independent prognostic indicators of colorectal SRCC patients. Married patients have better CSS and OS than unmarried patients. The findings of the current study require further study.
